# Drug pollution and pharmacotherapy in psychiatry: A “platypus” in the room

**DOI:** 10.1192/j.eurpsy.2020.32

**Published:** 2020-03-23

**Authors:** Unax Lertxundi, Rafael Hernández, Juan Medrano, Gorka Orive

**Affiliations:** 1 Bioaraba Health Research Institute, Osakidetza Basque Health Service, Araba Mental Health Network, Araba Psychiatric Hospital, Pharmacy Service, c/Alava 43, 01006 Vitoria-Gasteiz, Alava, Spain; 2 Internal Medicine Service, Araba Psychiatric Hospital, Araba Mental Health Network, c/Alava 43, 01006 Vitoria-Gasteiz, Alava, Spain; 3 Psychiatry Service, Bizkaia Mental Health Network, Vitoria-Gasteiz, Spain; 4 Biocruces Bizkaia Health Research Institute, Mental Health Network Research Group, Osakidetza, Bizkaia, Spain; 5 NanoBioCel Group, Laboratory of Pharmaceutics, School of Pharmacy, University of the Basque Country UPV/EHU, Paseo de la Universidad 7, Vitoria-Gasteiz 01006, Spain; 6 Biomedical Research Networking Centre in Bioengineering, Biomaterials, and Nanomedicine (CIBER-BBN), Vitoria-Gasteiz, Spain; 7 University Institute for Regenerative Medicine and Oral Implantology—UIRMI (UPV/EHU-Fundación Eduardo Anitua), Vitoria, Spain; 8 Singapore Eye Research Institute, The Academia, 20 College Road, Discovery Tower, Singapore, Singapore

**Keywords:** Drug therapy, environmental pollution, psychiatry

## Abstract

Preoccupation about potential deleterious effects of pharmaceuticals in the environment is growing fast. Psychiatric pharmaceuticals have received particular attention because of their increasing use and their potential impacts on many living beings due to their effects on phylogenetically highly conserved neuroendocrine systems. Recent studies that have shown that many pharmaceuticals (including psychotropics) bioaccumulate through the web food have raised this concern into new heights. As professionals working in the field of psychiatry and academia, we believe we are about to enter a new era with regard to pharmacotherapy. We estimate drug pollution will have a major impact on our daily practice in a way we are just starting to imagine. So far, this problem has largely been ignored by healthcare professionals, who are the ones prescribing and dispensing pharmaceuticals. We are convinced that increasing awareness among these professionals will be a key element to effectively fight against drug pollution.

Concern about potential deleterious effects of pharmaceuticals in the environment is rapidly growing worldwide [1]. Drug pollution has been considered as a “wicked problem” by the Dutch authorities: it is a complicated and diffuse problem that is characterized by scientific uncertainties, a large number of interested parties with different values and interests, and institutional complexity [2]. Once excreted by patient’s urine or feces, drugs, in the best of scenarios, are transported to sewage treatment plants, where actual widespread technology does not guarantee they are efficiently eliminated. Psychiatric pharmaceuticals have received particular attention because of their increasing use and their potential impacts on many living beings due to their effects on phylogenetically highly conserved neuroendocrine systems. Recent studies, however, have raised this preoccupation into new heights. Richmond et al. showed that many pharmaceuticals (including psychotropics) bioaccumulate through the food web, estimating that platypuses in Australia receive as much as half of the human antidepressant dose daily [3].

Nowadays, despite concerns may arise in the environmental risk assessment (ERA) of the drug presented to the European Medicines Agency (e.g., asenapine is believed to “have potential endocrine disruption properties [4]” and vortioxetine “is expected to pose a risk for the environment [5]”), environmental aspects are not considered in the global risk–benefit assessment of drugs for human use. Besides, these ERAs only became mandatory in 2005, so abundant knowledge gaps about many drugs which are extensively used in routine clinical practice still exist.

As professionals working in the field of psychiatry and academia, we believe we are about to enter a new era with regard to pharmacotherapy. We estimate drug pollution will have a major impact on our daily practice in a way we are just starting to imagine. Eco-sustainable or green prescribing has already been proposed by some authors, although not pertaining to healthcare [6]. For example, oxacepam, a drug that would be considered a wise choice for elderly people because the benign pharmacokinetic profile (it is eliminated via glucuronidation), persists without biodegradation in the bottom of lakes for decades [7]. Another relevant consequence may affect patients, in the way they may feel guilty about taking certain drugs. For instance, a depressed patient could become even more depressed when he hears about potential consequences of the drug he is excreting with his urine. Taking environmentally unfavorable medication could further add to the stigma of a mental illness. Additionally, psychiatric drugs are not popular even among people who could benefit from them; therefore, the awareness that a drug can have a deleterious impact on the environment can increase some patient’s reluctance toward psychopharmacological remedies. On the other hand, ecotoxicological aspects of psychiatric pharmaceuticals could transform into a potent argument for the antipsychiatry movement [8] and hospitals, could be banned or restricted near ecosystem reserves, or may even become a “not-in-my-backyard” institution.

The unwanted effects of drugs on ecosystems are gaining momentum, at a speed, that soon, pressure to include environmental risk aspects on the benefit/risk ratio of new drug approvals (already mandatory for veterinary products) will increase. In addition, ecotoxicological concerns may affect not only the drug but also the whole medicine itself. Think about inhaled loxapine, a recent marketed antipsychotic for the treatment of agitation, which necessitates that one lithium battery is discarded after each inhalation. Besides, in the case of Abilify Mycite, an electronic circuit is excreted with each ingested capsule.

As it occurs with ordinary pharmacovigilance, approved drugs could be withdrawn from the market because of eco-pharmacovigilance issues. Restricting the number of patients to be treated with certain drugs could become a reality. Moreover, less biodegradable drugs could become more expensive, and the pharmaceutical industry could be forced to develop “green by design” drugs, which are more biodegradable. Of course, a favorable environmental profile can become an issue in drug promotion, as a “favorable metabolic (or QTc, extrapyramidal) profile” has previously been raised to highlight a drug’s excellence.

So far, this problem has been addressed and discussed by chemists, environmentalists, and biologists, but has largely been ignored by healthcare professionals, who are the ones prescribing and dispensing pharmaceuticals. In fact, as far as we are concerned, issues about drug pollution are nowadays generally ignored in Pharmacy and Medicine faculties. In line with the recommendations given by the Strategic Approach of the European Commission, we are convinced that increasing awareness among these professionals will be a key element to effectively fight against drug pollution. We believe neglecting drug pollution aspects could contribute to the discredit of medicine, healthcare, and science in general. It is time to realize we have a platypus in the room.
